# Supplementary material for: Wet-Spun Chitosan–Sodium Caseinate Fibers for Biomedicine: From Spinning Process to Physical Properties
Source: Int J Mol Sci. 2024 Feb 1;25(3):1768. doi: 10.3390/ijms25031768 (PMC10855522; doi:10.3390/ijms25031768)
Supplement: Supplementary file 1 [file ijms-25-01768-s001.zip › ijms-2791135-supplementary.pdf]

Supplementary Materials

Wet-Spun Chitosan-Sodium Caseinate Fibers for Biomedicine: from Spinning Process to Physical Properties

Hazel Peniche [1,2], Ivy Ann Razonado [1], Pierre Alcouffe [1], Guillaume Sudre [1], Carlos Peniche [2,3], Anayancy Osorio-Madrazo [3,4*], Laurent David [1*]

[1] Ingénierie des Matériaux Polymères (IMP), Universite Claude Bernard Lyon 1, INSA de Lyon, Universite J. Monnet, CNRS, UMR 5223, 69622 Villeurbanne Cedex, France ; icrazonado@up.edu.ph (I.A.R.) ; pierre.alcouffe@univ-lyon1.fr (P.A.) ; guillaume.sudre@univ-lyon1.fr (G.S.)

[2] Biomaterials Center, Faculty of Chemistry, University of Havana, 10600 Havana, Cuba; hazel@biomat.uh.cu (H.P.); peniche@fq.uh.cu (C.P.)

[3] Friedrich Schiller University of Jena, Laboratory of Organic and Macromolecular Chemistry; Jena Center for
Soft Matter JCSM; and Center for Energy and Environmental Chemistry Jena CEEC, 07743 Jena, Germany

[4] University of Bayreuth, Laboratory of Organ Printing, 95447 Bayreuth, Germany

* Correspondence: anayancy.osorio.madrazo@uni-jena.de (A.O.-M.); laurent.david@univ-lyon1.fr (L.D.)

Table S1. Formulation and pH of mixed chitosan/sodium caseinate collodions for processing route R1.

Formulation Parameter		Pure Chitosan	CTS-R1 Cas 0.04%	CTS-R1 Cas 0.4%	CTS-R1 Cas 1.9%	CTS-R1 Cas 3.7%	CTS-R1 Cas 7.2%
m_{chi} (g)	Mass of engaged chitosan	3	0.75	0.75	0.75	0.75	0.75
m_{Cas} (mg)	Mass of engaged Sodium Caseinate	0	9.6	95.5	477.6	955.1	1910.2
m_{H2O} (g)	Mass of water	47.96	23.98	23.98	23.980	23.98	23.98
pH	After collodion mixing	4.74	4.90	5.12	5.18	5.32	5.94
c_{chi} (%w/w)	Mass fraction of chitosan	3.03	3.03	3.03	3.03	3.03	3.03
c_1=c_{cas} (%w/w)	Mass fraction of sodium Caseinate	0	0.04	0.4	1.9	3.7	7.2
r_1= m_{Cas}/ m_{chi}	Caseinate/chitosan mass ratio	0	0.013	0.13	0.64	1.27	2.55

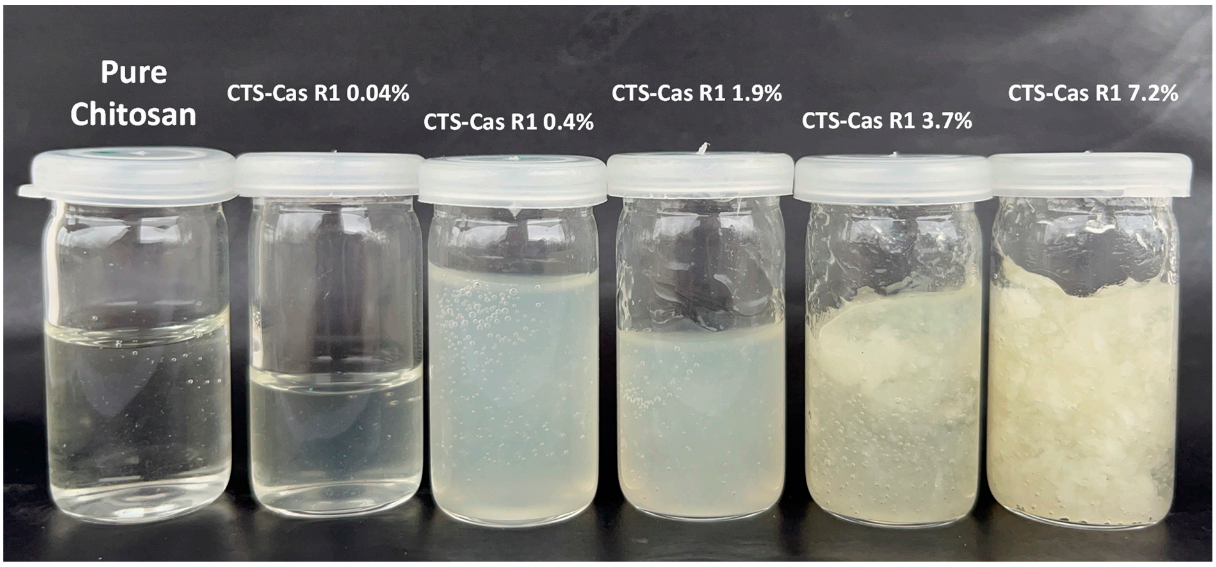

Figure S1. Aspect of pure chitosan solution and mixed collodions prepared at different sodium caseinate concentrations c_1=c_{cas}, ranging from 0.04% w/w to 7.2 %w/w, and constant chitosan concentration of c_{chi}~3%. The photograph was taken 12h after mixing. At a sodium caseinate concentration of 7.2% w/w, the system is a gel in this vial flow experiment.
